# Kinetics of the Photoexcited States in Thin Films of Metallo-Supramolecular Polymers With Ditopic Thiophene-Bridged Terpyridine Ligands

**DOI:** 10.3389/fchem.2021.766121

**Published:** 2022-01-20

**Authors:** Miroslav Menšík, David Rais, Muhammed Arshad Thottappali, Pinar Güloğlu, Petr Toman, Jiří Vohlídal, Jiří Pfleger

**Affiliations:** ^1^ Institute of Macromolecular Chemistry, Czech Academy of Sciences, Prague, Czechia; ^2^ Faculty of Mathematics and Physics, Charles University, Prague, Czechia; ^3^ Department of Physical and Macromolecular Chemistry, Faculty of Science, Charles University, Prague, Czechia

**Keywords:** metallo-supramolecular polymers, transient absorption spectroscopy, singlet and triplet excitons, time-dependent diffusion coefficient, polythiophene

## Abstract

Managing the excited-state decay by a supramolecular structure is a crucial issue for organic photovoltaics. We show that in thin films of metallo-supramolecular polymers made of bis(terpyridine-4′-yl)terthiophenes and $Zn^{2+}$ coupling ions, the photoexcited states generated by ultrashort laser pulses at the wavelength of 440 nm decay by the bi-molecular annihilation predominantly controlled by the Förster transfer between singlet states. During this bi-molecular annihilation of singlet states, intermediate hot triplet pairs are formed, which subsequently dissociate into long-living diffusing triplet states. It explains a significant shortening of the triplet state rise time with increasing pump fluence. The diffusion coefficient of triplets showed power-law time dependence, with its exponent proportional to the pump fluence, decreasing thus the diffusivity of triplets.

## Introduction

Metallo-supramolecular polymers (MSPs) are composed of defined simple or oligomeric organic molecules capped with chelating end-groups (referred to as unimers) that spontaneously assemble into polymer chains by coordination to metal ions (dubbed ion couplers) (Ciferri, 2002). The ideal MSP should exhibit high thermodynamic stability under operational conditions, but it should be kinetically labile (should significantly dissociate) in solutions and/or at elevated temperature under processing conditions (Constable et al., 1994). Kinetic lability gives to MSPs processing advantages, easier control of the morphology of thin films, and multilayered structures and opens up new possibilities of post-synthesis modifications and tailoring their properties. Polymers showing this so-called constitutional dynamics are referred to as dynamers (Lehn, 2005). Dynamics of MSP chains also allow their structure healing by exchanging and/or reshuffling of their unimeric constituents and/or ion couplers.

During the last decade, MSPs underwent tremendous development because of their unique electronic, photonic, magnetic, and catalytic properties (Hissler, 2019). MSPs containing π-conjugated building blocks gained interest as materials for optoelectronic devices, covering light to electricity conversion, and for polymer light-emitting diodes (PLEDs) (Hissler, 2019). Recent finding of an efficient singlet fission process in thin films of MSP based on α,ω−bis(tpy)terthiophene unimers assembled with Zn^2+^ ion couplers (Rais et al., 2017b) shows that some MSPs could also be potentially exploited for better light harvesting in photovoltaics. The supramolecular approach to polymer chemistry evidently meets the actual needs of new functional materials for advanced electronics. Linear conjugated MSPs described in the literature mostly comprise α,ω−bis(tpy) unimers (tpy stands for 2,2′,6′,2″-terpyridin-4′-yl end-group) with oligo (*p*-arylenevinylene) (El-Ghayoury et al., 2002), oligo (*p*-aryleneethynylene) (Burnworth et al., 2008), oligo(fluorene) (Chen and Lin, 2007; Hrma et al., 2017), oligo (phenylene) (Li et al., 2016; Winter and Schubert, 2016), or co-oligomeric (Chiper et al., 2008; Zhang et al., 2019) central units linked into MSP chains *via* coordination to Zn^2+^, Co^2+^, Fe^2+^, La^3+^, or Eu^3+^ ion couplers (Hissler, 2019). Specific facial-meridian coordination of tridentate terpyridine end-groups to ion couplers (Zhang et al., 2019) gives MSPs well-defined stereochemistry, which is important for reproducible preparation of functional materials. This feature is absent in many other MSPs (Burnworth et al., 2007).

The kinetics of photoexcited states in α,ω−bis(tpy)terthiophenes (unimer T) and their MSPs with Zn^2+^ ion couplers (PT) were measured using pump-probe transient absorption spectroscopy. In solutions, their photophysical properties were found to be strongly influenced by chain dynamics. Relaxation processes running in photoexcited molecules of these unimers and MSPs were identified and characterized, and the impact of disturbed coplanarity of adjacent rings (dihedral angles between planes of rings due to the attached hexyl side groups) on these processes was shown (Rais et al., 2015). Two different physical processes were found in thin films of PT depending on the excitation wavelength (Rais et al., 2017b). Using the wavelength 332 nm, the system showed a fast singlet fission (SF) process with a time constant t= 160 fs . At a longer timescale, the formed triplet state population followed a slow power-law decay. The fast bi-molecular annihilation of singlet excitons was deduced from the explicit dependence of the decay rates on the pump pulse intensity. On the other hand, when these PT thin films were photoexcited at the wavelength 440 nm, no sign of the SF process has been found. The singlet excitons showed a power-law decay rate dependent on the pump pulse fluence. It indicated the role of bi-molecular collision events in the singlet annihilation process. At longer times after photoexcitation, the transient absorption spectra proved a well-discerned formation of excited state absorption (ESA) assigned to triplet states. Interestingly, the rise time of this ESA formation was strongly dependent on the pump fluence, ranging from ca 20 ps at low pump fluence down to ca 3 ps at high pump fluence. These triplet excitations also proved power-law decay. Although the evolution of singlet and triplet exciton populations was experimentally well-characterized in the study by Rais et al., (2017b), the origin of the bi-molecular singlet annihilation and subsequent triplet formation and decay remained unexplained. Meanwhile, we have elaborated a novel mathematical technique (Menšík et al., 2018a; Rais et al., 2018; Menšík et al., 2019), which makes the determination of the time-resolved diffusion coefficient from the transient absorption kinetics of photoexcited species possible. In this article, we applied the developed procedure on the previously acquired experimental data (Rais et al., 2017b) and proved that it is capable of elucidating in detail the process of the bi-molecular singlet annihilation and the subsequent formation and de-excitation of the triplet states.

## Experimental

**FIGURE 1:**
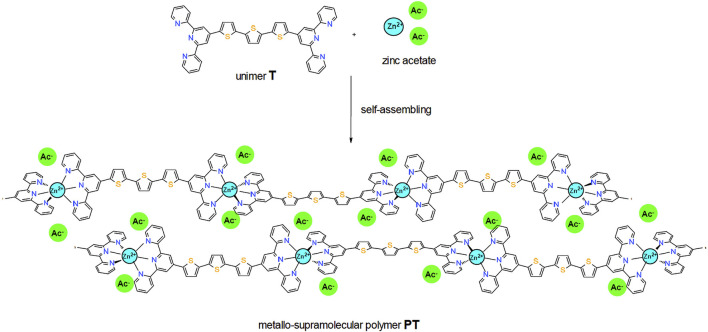
Formation of supramolecular structure of thin films of the metallo-supramolecular polymer PT.

The material chemistry, thin film preparation, X-ray diffractograms (XRDs) of PT films, and transient absorption (TA) spectroscopy experiment were described in details by Rais et al. (2017b) and the references therein. Here, we just briefly mention the following: 1) The procedure of synthesis of compounds of α,ω-bis(tpy)terthiophene (T) and its zinc-bridged supramolecular polymer PT (to keep the same notation as given by Rais et al. (2017b) (cf. Figure 1) in a powder form was described by Svoboda et al. (2011), Bláhová et al. (2014). 2) PT films deposited on 4 × 5 cm rectangular quartz substrates were prepared from a hexafluoroisopropanol solution of an equimolar mixture of T and zinc acetate by spin-casting at 3,000 rpm. 3) The XRD of the as-prepared PT powder samples of PT showed a dominant peak at about 26° , which corresponded to the inter-planar distance of 3.4 Å and a band of amorphous halo. This indicated a highly ordered state of 1d anisotropy of the PT samples stacked in the face-to-face direction. 4) For the TA spectroscopy, described in detail by Rais et al., (2015), we used the linearly polarized excitation and detection laser pulses at the so-called “magic” angle α = 54.7° to avoid the anisotropy effects induced by the photoexcitation (Fleming et al., 1976).

The transient differential absorption spectra ΔA(λ,t) were monitored in the interval from 550 to 800 nm (corresponding to the ESA of the PT films photoexcited by a 440-nm pump pulse) and analyzed using the following equation (Rais et al., 2017b):

$$\Delta A\left(\lambda ,t\right)=a_{S}\left(\lambda \right)c_{S}\left(t\right)+a_{T}\left(\lambda \right)c_{T}\left(t\right).$$ (1)

Here, $a_{S}\left(\lambda \right)$ and $a_{T}\left(\lambda \right)$ are known basis spectral functions of singlets and triplets, respectively. Because they were obtained up to a constant scaling factor, the evolutions of singlets $\left(c_{S}\left(t\right)\right)$ and triplets $\left(c_{T}\left(t\right)\right)$ could be determined only in arbitrary units (Rais et al., 2017b). However, their population fractions with respect to the total number of unimer units can be recalculated from known kinetics of the ground-state bleach at early times and the steady-state absorption spectrum as it is shown below.

## Singlet Exciton Population Kinetics

**FIGURE 2:**
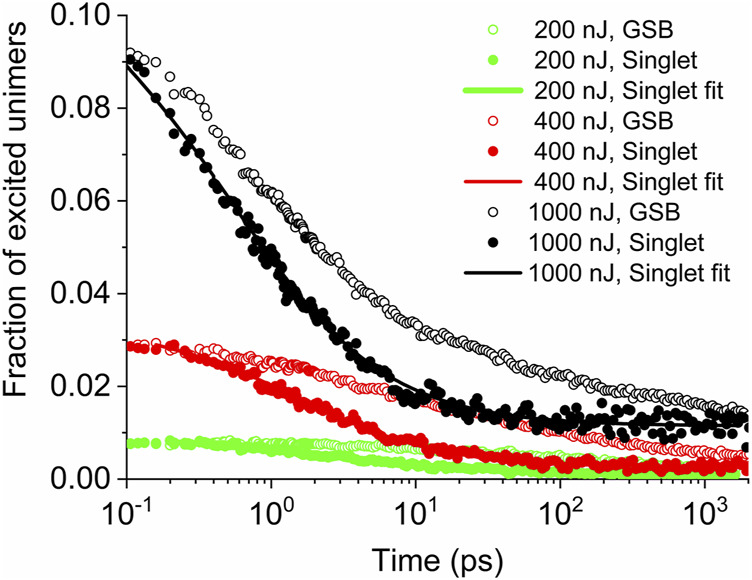
Time evolution of the singlet exciton population (full symbols) and of the ground-state bleach (open symbols) for different pump fluences indicated in the legend. Solid lines represent the best fits according to Eq. 2.

In Figure 2, we show the evolutions of the populations of the singlet excitons in the PT thin film after excitation by the pump pulse at the wavelength 440 nm, as obtained from Eq. 1. Note that the singlet population equals the ground-state bleach immediately after the excitation because only singlets are directly created by the pump pulse. It is known that the probe at 500 nm provides a nearly exclusively ground-state bleach (GSB) signal, that is, the sum of singlet and triplet excitons with equal weights, without an overlap with the positive ESA signal, which contributes to the spectrum from ca 550–800 nm. Therefore, the measured intensities of the GSB signal can be considered directly proportional to the sum of the concentrations of singlet and triplet excitons. Considering that a singlet exciton is located on a single unimeric unit due to relatively weak mutual interaction of the frontier orbitals of neighboring units, the ratio of the initial TA signal at 500 nm and the steady-state absorption at 500 nm directly shows the fraction of excited unimers in the sample. The absolute values of the singlet populations were obtained from their relative values in Eq. 1, assuming that exclusively singlet excitons contributed to the GSB signal immediately after the photoexcitation.

The obtained time dependences of the fraction of singlet excited unimers S(t) , referred to as singlet exciton population further in the text, were fitted for different pump fluences according to the following formula

$$S\left(t\right)=\frac{A}{1+B{\left(t-0.2\right)}^{\delta }}+C,$$ (2)

where time t is expressed in ps, and the values of the parameters *A, B, C*, and *δ* for different pump fluences are shown in Table 1.

**TABLE 1:** Parameters from Eq. 2 obtained by fitting experimental data for different pump fluences.

Pump fluence F[nJ]	*A*	*B*	*C*	δ
200	0.0073	0.37	0.00079	0.75
400	0.026	0.58	0.026	0.90
1,000	0.082	1.35	0.012	0.85

It is seen from Figure 2 that the term $\frac{A}{1+B{\left(t-0.2\right)}^{\delta }}$ represents the annihilation of singlets on the time scale of hundreds of picoseconds, where the decay of singlets is dominant, while the “additive term” in the right-hand side of Eq. 2 corresponds to the residual longer-living singlet population decaying on time scale significantly longer than nanoseconds. Since the time course at longer times could not be reliably distinguished due to the poor signal-to-noise ratio, it was approximated by constant. Data in Figure 2 also show that the singlet decay rate increases with the pump fluence. This indicates that the decay of the singlet exciton volume density $s\left(t\right)=N_{0}S\left(t\right)$ (here $N_{0}$ is the unimer volume density) is controlled by the mutual exciton–exciton annihilation process according to the equation (Engel et al., 2006; Menšík et al., 2018a)

$$\frac{\partial }{\partial t}s\left(t\right)=-\gamma \left(t\right)s{\left(t\right)}^{2}.$$ (3)

Solution of which is a function

$$s\left(t\right)=\frac{s\left(0\right)}{1+s\left(0\right)\int_{0}^{t}\gamma \left(\tau \right)d\tau }.$$ (4)

In Eqs 3, 4, γ(t) determines the rate of bimolecular collisions leading to exciton annihilation. The bimolecular mechanism can be experimentally confirmed by the power-law dependence of the population decay, easily seen when plotting it in the log–log scale (Engel et al., 2006; Marciniak et al., 2011), and by the explicit dependence of the population decay rate on the excitation pump fluence. Both these effects were found by Rais et al. (2017b). The linear decay term (originated e.g. in the exciton annihilation by fluorescence emission) could be also added, but it does not play a role within the time scale discussed in this article. It would have an observable effect only at longer times for sufficiently low singlet population and could be seen when plotting time course of the population decay in the semilogarithmic scale, which was not the case of the discussed experimental data. We see that the algebraic structure of Eq. 4 is similar to the term $\frac{A}{1+B{\left(t-0.2\right)}^{\delta }}$ in Eq. 2 that dominantly describes the singlet decay, before the long-tail dependence (represented by the additive term) becomes relevant. Eq. 4 can also be equivalently cast into the form

$$\frac{1}{s\left(t\right)}-\frac{1}{s\left(0\right)}=\overset{t}{\underset{0}{\int }}\gamma \left(\tau \right)d\tau .$$ (5)

Eq. 5 can be also expressed in terms of the singlet exciton population S(t) as

$$\frac{1}{S\left(t\right)}-\frac{1}{S\left(0\right)}=N_{0}\overset{t}{\underset{0}{\int }}\gamma \left(\tau \right)d\tau .$$ (6)

Substituting S(t) from Eq. 2 for to the left-hand side of Eq. 6, we directly obtain the kinetics of the integral of the rate $N_{0}\gamma \left(t\right)$ of the exciton–exciton annihilation. This annihilation can, in principle, be controlled by either the Förster transfer or the diffusion process.

The Förster transfer is controlled by the long-range dipole–dipole interaction of two, in this case both excited, unimers. Within the discussed recombination process one of them becomes de-excited, while the other one is excited to a higher excited state followed by the subsequent fast Kasha de-excitation from higher to lower excited state or, alternatively, by singlet fission. As a result, there is either only one unimer remaining in the singlet state or two unimers in the triplet state. Such a process can be formally expected due to a significant overlap between the luminescence spectrum and the excited state absorption (ESA) spectrum at wavelengths between 600 and 800 nm (cf. Figure 4-4 of Rais et al. (2017b)).

The diffusion (hopping) proceeds, in turn, by subsequent exciton hopping between the nearest neighbor molecules, facilitated by the overlap of their electronic orbitals. It is, in principle, supported by certain regularity of the arrangement of PT units in thin films characterized by the inter-planar distance of 3.4 Å. It allows getting excitations to the nearest distance where they can recombine.

**FIGURE 3:**
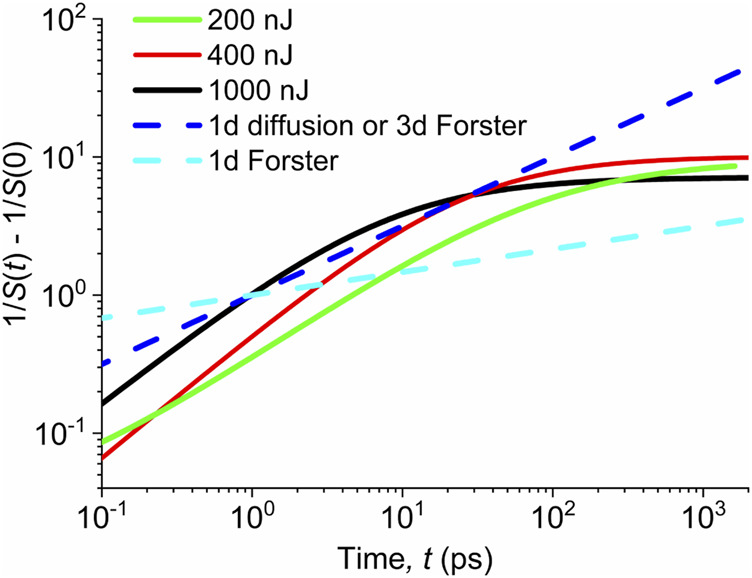
Solid lines—evolution of the inverse of the singlet exciton population estimated from the TA data measured with various pump fluences indicated in the legend. Dashed lines—power-law profiles of the exciton–exciton annihilation controlled by either 1d diffusion with the time-independent diffusion coefficient or 3d Förster transfer (light blue) or 1d Förster transfer (dark blue). The initial singlet populations are normalized to unity (independent on the pre-factors in Eqs 5, 6) for all pump fluences.

The dependences $\frac{1}{S\left(t\right)}-\frac{1}{S\left(0\right)}$ obtained from fitting the evolution of singlet population S(t) , according to Eq. 2, were plotted in Figure 3, with the singlet populations normalized to unity at early times for all pump fluences. We compared them with theoretical dependences calculated according to Eq. 6, assuming that the excitation annihilation is controlled by either 1d diffusion with a constant diffusion coefficient ( $N_{0}\overset{t}{\underset{0}{\int }}\gamma \left(\tau \right)d\tau ∼t^{1/2}$ , Ref (Engel et al., 2006)), 3d Förster transfer $\left(N_{0}\overset{t}{\underset{0}{\int }}\gamma \left(\tau \right)d\tau ∼t^{1/2}\right)$ or 1d Förster transfer $\left(N_{0}\overset{t}{\underset{0}{\int }}\gamma \left(\tau \right)d\tau ∼t^{1/6}\right)$ (Marciniak et al., 2011).

### Interpretation of the Singlet Exciton Annihilation by the Förster Transfer

If the singlet annihilation is interpreted within the Förster transfer model, the slopes of the dependences shown in Figure 3 suggest that for times up to ca 10 ps, the process is rather of 3d nature, while it takes a 1d character at longer times. This is not in contradiction with the physical intuition. Namely, respective transition dipole moments for the absorption or luminescence are probably aligned parallel (or antiparallel) in the anisotropic 1d structure. If the vector $\vec{r}$ connecting two annihilating excitations is parallel (or antiparallel) to the respective transition dipole moments, their dipole–dipole interaction is two times stronger than in the case where it is perpendicular to the respective transition dipole moments. The rate of the second-order Förster transfer is then four times higher. Consequently, excitations in such directions become statistically more depleted after some time, and the space distribution of singlet states becomes inhomogenous forming excitation domains with their connecting vector $\vec{r}$ perpendicular to the respective transition dipole moments. It will promote the annihilation process by the 1d Förster transfer mechanism at longer times.

### Interpretation of the Singlet Exciton Annihilation by Diffusion

On the microscopic level, the diffusion of singlet excitons is controlled by hopping probability between nearest neighbor unimers. Due to face-to-face molecular stacking, such a process is dominantly of 1d nature. In Figure 3, we see that the data can be well-interpreted within the concept of the 1d model with the diffusion coefficient gradually decreasing in time, but the 1d model with the time-independent diffusion coefficient fails. For this case, we can relate the term $N_{0}\overset{t}{\underset{0}{\int }}\gamma \left(\tau \right)d\tau$ from the right-hand side of Eq. 6 with the 1d time-dependent coefficient $D_{1d}\left(t\right)$ (Menšík et al., 2018a; Rais et al., 2018; Menšík et al., 2019) as

$$\frac{1}{S\left(t\right)}-\frac{1}{S\left(0\right)}=N_{0}\overset{t}{\underset{0}{\int }}\gamma \left(\tau \right)d\tau =\frac{1}{a}\sqrt{\frac{32\int_{0}^{t}D_{1d}\left(s\right)ds}{\pi }}$$ (7)

or equivalently

$$D_{1d}\left(t\right)=\frac{\pi }{16}a^{2}\gamma \left(t\right)\overset{t}{\underset{0}{\int }}\gamma \left(\tau \right)d\tau =-\frac{\pi }{16}a^{2}\left(\frac{1}{S\left(t\right)}-\frac{1}{S\left(0\right)}\right)\frac{1}{S{\left(t\right)}^{2}}\frac{\partial }{\partial t}S\left(t\right).$$ (8)

**FIGURE 4:**
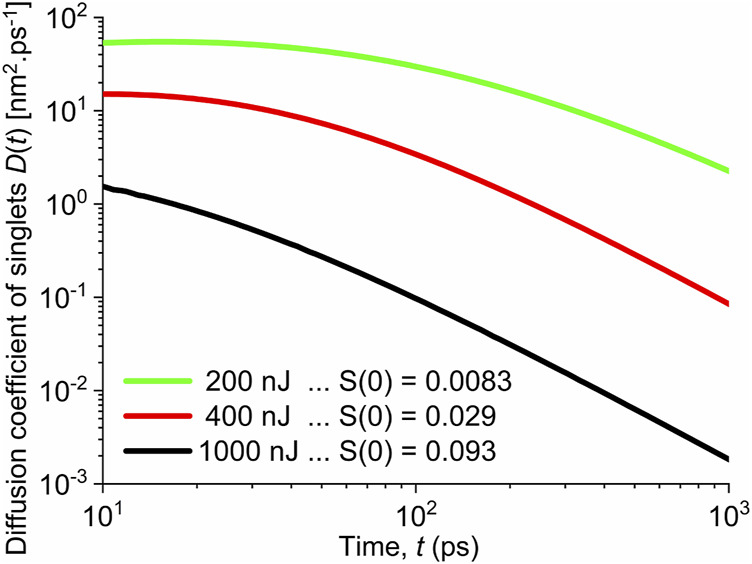
Diffusion coefficient of singlet excitons assuming the singlet–singlet annihilation is fully controlled by the singlet exciton diffusion. Shown for different pump fluences and corresponding initial excitation populations marked in the legend.

In Eq. 7, a denotes the distance between the hopping sites. In Figure 3, we see that for long times, the value of $\left(\frac{1}{S\left(t\right)}-\frac{1}{S\left(0\right)}\right)$ almost saturates (the saturation is reached earlier for higher pump fluences). Consequently, also, the value of $\overset{t}{\underset{0}{\int }}D_{1d}\left(s\right)ds$ in Eq. 7 almost saturates. The integral $\overset{t}{\underset{0}{\int }}D_{1d}\left(s\right)ds$ can converge only if the singlet diffusion coefficient $D_{1d}\left(t\right)$ decays in time. In Figure 4, we show the time evolution of the diffusion coefficient $D_{1d}\left(t\right)$ obtained from Eq. 8 using the smooth profile of the singlet population decay obtained by fitting experimental data according to Eq. 2. The presented time dependences in Figure 4 are shown only for times longer than 10 ps when the full thermalization of exciton population is guaranteed and description within diffusive motion can be adopted. We observe two important properties: First, with increasing pump fluence, that is, with increasing concentration of initially excited unimers, the diffusion coefficient decreases more rapidly in time. While at low initial excitation densities the diffusion coefficient changes relatively little in time, for the high initial excitation density of ca S(0)≅0.093 , the diffusion coefficient decreases by ca 3 orders of magnitude between 10 and 1,000 ps. Such behavior could be expected as the diffusing excitons that are closest to each other recombine first, depleting their population locally and, as a result, the exciton population becomes modulated locally in space. The excitation distribution becomes, thus, more localized, and the diffusion coefficient of singlet excitations decreases. On the other hand, for low initial excitation population S(0)≅0.0083 , we obtained very high initial value of the diffusion coefficient of singlets $D_{1d}\left(0\right)\approx 54\;{\text{nm}}^{2}\;{\text{ps}}^{-1}$ . This is in contradiction to typical values of a few ${\text{nm}}^{2}\;{\text{ps}}^{-1}\;$ found in polymers (Rais et al., 2017a). Realizing that for times *t* up to ca 10 ps $D_{1d}\left(t\right)\leq D_{1d}\left(0\right)$ , we can estimate the mean diffusion distance at early times. Namely, within ca 0.1 ps, that is, in the time scale of intramolecular vibronic decoherence, the diffusion length would be $\sqrt{2D_{1d}\left(0\right)t}\approx 3.3\;\text{nm}$ , corresponding to ca 10 unimers stacked in the face-to-face direction. At times of 1 ps, typical for the energy transfer between the nearest molecules, the diffusion length would be $\sqrt{2D_{1d}\left(0\right)t}\approx 10\;\text{nm},$ covering ca 30 unimers stacked in the face-to-face direction, which is not realistic. Such large diffusion distance comes from the fact that in the annihilation process, the effective radius of exciton–exciton interaction exceeds the dimension of several unimers. But, it cannot happen if only hopping between the nearest unimers is considered. Instead, it requires the participation of the long-range Förster transfer, where the Förster radius typically exceeds several nanometers.

We conjecture from the abovementioned discussion that the long-range Förster energy transfer process will be dominant. The diffusion-controlled annihilation participates only as a secondary additive process, but becomes important when the initial excitation population is high.

## Triplet Exciton Population Kinetics

**FIGURE 5:**
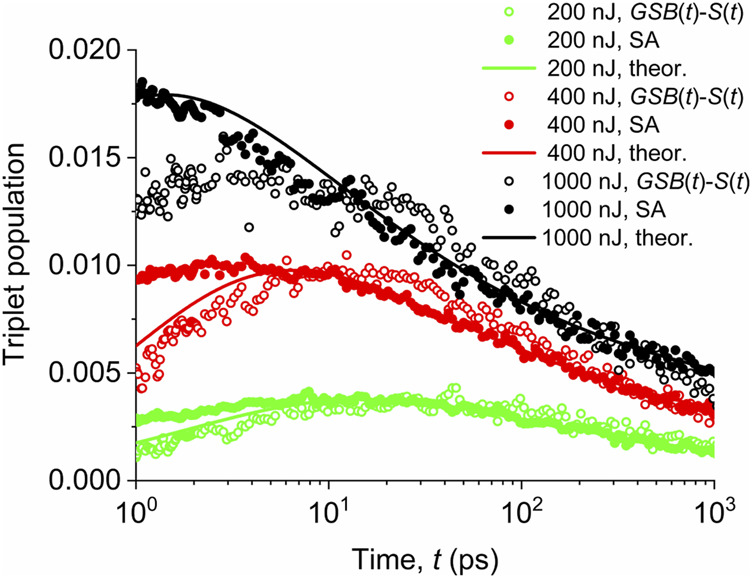
Evolution of the triplet exciton population for different pump fluences indicated in the legend. Empty symbols—values obtained by subtracting experimental data of ground-state bleach and singlet populations. Full symbols—data from the spectral analysis (SA) of transient absorption reported by Rais et al. (2017b). Solid lines—data calculated using a theoretical model based on the triplet generation by the singlet–singlet annihilation and the triplet population decay controlled by the triplet–triplet annihilation.

Experimental data of the triplet population decay for different pump fluences are shown in Figure 5. For the sake of clarity, we show them as two independent sets: 1) derived from Figure 2 by subtracting the ground-state bleached population GSB(t) and the singlet exciton population S(t) and 2) taken from the spectral analysis (SA) of transient kinetics reported by Rais et al. (2017b). In the first case, the ground-state bleached population GSB(t) was obtained from the ratio of the ΔA(λ,t) kinetics and the steady-state absorbance at 500 nm (GSB region); the singlet state population S(t) , which is proportional to $c_{S}\left(t\right)$ in Eq. 1, was normalized to the initial value at time t = 0 as S(0)=GSB(0) . The triplet population dependence was, thus, obtained partly from the kinetic trace at the GSB region and partly by the spectral decomposition of the transient absorption in the ESA region.

In the second case, the triplet population kinetics was directly obtained from Eq. 1, where it is proportional to $c_{T}\left(t\right)$ , and from known basis spectral functions of singlets and triplet excitons. As in this case, the triplet exciton population is exact up to a normalization constant; we normalized it to give identical values as the first set of the triplet excitation dependences for the delay time of 10 ps.

We see in Figure 5 that both methods provide identical profiles at longer times, when the excited-state manifold is already thermalized, while they differ for short times, when the hot-state kinetics of the excited-state manifold can play a role.

We also see that for low pump fluence of 200 nJ (initial singlet exciton population S(0)=0.0083 ), the triplet population T(t) initially increases with time. At a delay time of ca 20 ps, the triplet exciton population reaches the maximum value and decreases at longer times. We can see that with increasing intensity of the pump fluence, the T(t) reaches its maximum faster. For the pump fluence of 400 nJ (S(0)=0.029) , the maximum of the triplet population is reached at ca 5–10 ps, while for the pump fluence of 1,000 nJ (S(0)=0.093) , it is reached already within 1–3 ps. At longer times, the population decay asymptotically approaches the power-law dependence, with the exponent dependent on the pump fluence. Both of these facts indicate that the triplet population decay proceeds *via* the diffusion-controlled triplet–triplet annihilation.

We should note that the power-law decay is typically observed for diffusion processes (see discussion below). The rate of the triplet population decay depends on the pump pulse fluence, which also determines the initial density of triplet state population.

In the study by Rais et al. (2017b), it was shown that the SF process occurs after the excitation to the S_2_ state and not to the S_1_ state because the energy of the triplet pair is higher than the energy of S_1_. In reverse, the triplet–triplet annihilation can also yield partly S_1_ singlets. It would also explain the decreasing rate of the singlet population decay at very long times.

Based on the abovementioned discussion, the triplet population kinetics can be described by the following equation

$$\frac{\partial }{\partial t}T\left(t\right)=G\left(t\right)-\gamma_{1d}^{T}\left(t\right)T{\left(t\right)}^{2}.$$ (9)

Here, G(t) stands for the rate of triplet generation. As triplets are formed from the fast decaying singlet states, the function G(t) becomes very small at longer times. Below, we will discuss two possible ways of the triplet exciton formation from singlet excitons.

In the second term in Eq. 9, the term $\gamma_{1d}^{T}\left(t\right)$ represents the rate of the triplet–triplet annihilation. By fitting experimental data, we found that the power-law ansatz $\gamma_{1d}^{T}\left(t\right)=\gamma_{0}t^{-r}$ , with 0<r<1 , well-describes the long-time triplet power-law $\left(T\left(t\right)∼T_{0}t^{-q}\right)$ asymptote (cf. Figure 5). It can be simply checked that if the triplet decay in Eq. 9 is controlled only by the triplet–triplet annihilation (at longer times), we directly obtain for parameters of the fit the relation $0<q=-r+1=\gamma_{0}T_{0}<1$ . The exact analytical solution to Eq. 9 is unknown; however, we could easily find an upper limit for its solution $\bar{T}\left(t\right)$ . It coincides with the exact solution for an early time limit. Namely,

$$\bar{T}\left(t\right)=\overset{t}{\underset{0}{\int }}G\left(\tau \right)d\tau \;\mathrm{for}\;t\leq t_{1},$$ (10)

which asymptotically describes the early time “triplet pumping” when the triplet–triplet annihilation could be neglected due to their low populations.

For longer time limit, the experimental data approach the asymptote

$$\bar{T}\left(t\right)=T_{0}t^{-q}\;\text{for}\;t\geq t_{1},$$ (11)

which follows from Eq. 9 if the “pumping rate” G(t) is depleted.

The abovementioned two asymptotes describe the evolution at early and long-time limits, respectively. At the same time, it is evident that $T\left(t\right)≦\bar{T}\left(t\right)$ over the whole experimental time interval.

We can define the point $t_{1}$ as the time when these two asymptotes intersect. It can be shown that this point $t_{1}$ is located near the time when the triplet population takes the maximum value. As the time dependence of the triplet population is slowly varying the function around its maximum, we can derive $\frac{\partial }{\partial t}T\left(t_{1}\right)\approx 0$ . Then, from Eq. 9, we get $G\left(t_{1}\right)\approx \gamma_{1d}^{T}\left(t_{1}\right)T{\left(t_{1}\right)}^{2}$ . We can compare this value with the mean value of the rate G(t)  in the interval, $0\leq t\leq t_{1},\bar{G}\left(t\right)=\frac{1}{t_{1}}\overset{t_{1}}{\underset{0}{\int }}G\left(t\right)dt:$

$$\begin{array}{l} \frac{G\left(t_{1}\right)}{\frac{1}{t_{1}}\int_{0}^{t_{1}}G\left(t\right)dt}\approx \frac{\gamma_{1d}^{T}\left(t_{1}\right)T{\left(t_{1}\right)}^{2}}{\frac{1}{t_{1}}\int_{0}^{t_{1}}G\left(t\right)dt}=\frac{\gamma_{1d}^{T}\left(t_{1}\right)T{\left(t_{1}\right)}^{2}}{\frac{1}{t_{1}}\bar{T}\left(t_{1}\right)}=t_{1}\gamma_{1d}^{T}\left(t_{1}\right)\bar{T}\left(t_{1}\right){\left(\frac{T\left(t_{1}\right)}{\bar{T}\left(t_{1}\right)}\right)}^{2}=\\ =t_{1}\gamma_{0}t_{1}^{-r}T_{0}t_{1}^{-q}{\left(\frac{T\left(t_{1}\right)}{\bar{T}\left(t_{1}\right)}\right)}^{2}=q{\left(\frac{T\left(t_{1}\right)}{\bar{T}\left(t_{1}\right)}\right)}^{2}<1. \end{array}$$ (12)

We could see that the rate of triplet population generation at time *t*_1_ is already lower than the mean rate of triplet generation in the interval (0, *t*_1_). Thus, near the maximum of the triplet population also the “pumping rate” of triplet states decreases. It also proves that the triplet generation is directly bound to singlet exciton population decay.

The fact that near the point of the maximum of triplet population, the function $\overset{t}{\underset{0}{\int }}G\left(\tau \right)d\tau$ should saturate can be derived also from the analytical examination of the second derivative of the triplet population.

Taking into account the function used for fitting experimental data for the triplet annihilation rate $\gamma_{1d}^{T}\left(t\right)=\gamma_{0}t^{-r}$ (see the text above) and differentiating Eq. 9, we get directly

$$\begin{array}{l} \frac{\partial^{2}}{\partial t^{2}}T\left(t\right)=\frac{\partial }{\partial t}G\left(t\right)-\left(\frac{\partial }{\partial t}\gamma_{1d}^{T}\left(t\right)\right)T{\left(t\right)}^{2}-2\gamma_{1d}^{T}\left(t\right)T\left(t\right)\frac{\partial }{\partial t}T\left(t\right)=\frac{\partial }{\partial t}G\left(t\right)-\left(\frac{\partial }{\partial t}\gamma_{1d}^{T}\left(t\right)\right)T{\left(t\right)}^{2}\\ -2\gamma_{1d}^{T}\left(t\right)T\left(t\right)\left(G\left(t\right)-\gamma_{1d}^{T}\left(t\right)T{\left(t\right)}^{2}\right)=\left\{\frac{\partial }{\partial t}G\left(t\right)-2\gamma_{1d}^{T}\left(t\right)T\left(t\right)G\left(t\right)\right\}+T{\left(t\right)}^{2}\left\{-\frac{\partial }{\partial t}\gamma_{1d}^{T}\left(t\right)+2T\left(t\right){\left(\gamma_{1d}^{T}\left(t\right)\right)}^{2}\right\}\\ =\left\{\frac{\partial }{\partial t}G\left(t\right)-2\gamma_{0}t^{-r}T\left(t\right)G\left(t\right)\right\}+\gamma_{0}T{\left(t\right)}^{2}\left\{rt^{-r-1}+2\gamma_{0}T\left(t\right)t^{-2r}\right\} \end{array}.$$ (13)

We see that the second derivative $\frac{\partial^{2}}{\partial t^{2}}T\left(t\right)$ consists of two terms denoted by curly brackets {}. The first term, containing the monotonically decreasing pumping rate G(t) , is always negative and thus contributes to the “concavity” of the time course of the triplet population, while the second term, formed only by the triplet–triplet annihilation, is always positive and thus contributes to the convexity of the time dependence of the triplet population. The first term is dominant for short times; however, when the integral $\;\overset{t}{\underset{0}{\int }}G\left(\tau \right)d\tau$ saturates, then also the monotonically decreasing function G(t)→0 and also $\frac{\partial }{\partial t}G\left(t\right)\to 0$ . Then, the first term in Eq. 13 will disappear, and the time course of the triplet population will attain the convex profile.

The experimental data show that not only the position of the maximum of the triplet population but also the transition time between the concavity and convexity of the triplet population time course shifts to shorter time with increasing pump fluence. It means that also the saturation of the “pumping rate integral” $\overset{t}{\underset{0}{\int }}G\left(\tau \right)d\tau$ should occur at shorter time when the pump fluence is increased. This conjecture will be also used for the analysis of the mechanism of the triplet exciton generation below. We will discuss two physical mechanisms of the triplet formation: 1) inter-system crossing and 2) singlet–singlet collision.

### Triplet Exciton Formation by Inter-System Crossing

Here, we assume that the spin-orbital coupling will allow the singlet-to-triplet transition *via* inter-system crossing (Sun et al., 2021). The rate of the formation of the triplet population T(t) would then be proportional to the instant value of the singlet exciton population S(t) .

G(t)=αS(t), (14)

where α is a constant independent on the exciton population, and thus also the initial pump fluence. After integration over time, we get

$$\overset{t}{\underset{0}{\int }}G\left(\tau \right)d\tau =\;\alpha \overset{t}{\underset{0}{\int }}S\left(\tau \right)d\tau ,$$ (15)

so that the rate of the depletion of the “pumping rate” generated by the inter-system crossing can be obtained by analyzing the time evolution of $\overset{t}{\underset{0}{\int }}S\left(\tau \right)\mathrm{d\tau}$ from the singlet state kinetics, particularly its saturation onset.

### Triplet Formation by the Singlet–Singlet Annihilation

**FIGURE 6:**
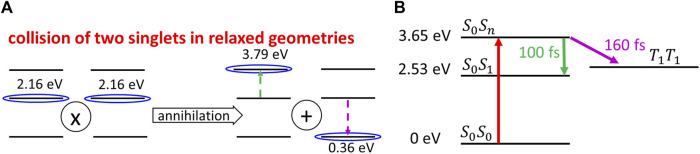
**(A)** Scheme of the singlet–singlet annihilation process, which can be described as a simultaneous virtual ESA (indicated by the green dashed arrow) and the virtual luminescence (shown by the violet dashed arrow). **(B)** Internal conversion to the $S_{1}$ state by the Kasha process during ca 100 fs after the excitation to the higher excited state $S_{n}$ and simultaneous singlet fission process with a time constant ca 160 fs (Rais et al., 2017b).

The possibility of this process can be justified by the following reasons. In the study by Rais et al. (2017b), it was shown that the maximum absorption from the ground state takes place at 2.53 eV, while the maximum of the emission takes place at 1.80 eV. From the Stokes shift energy 0.73 eV, we can estimate the energy of $S_{1}$ in the relaxed geometry with respect to that of $S_{0}$ for equilibrium geometry to be ca 2.16 eV. The maximum of the observed $S_{1}$ ESA at 1.63 eV falls within its fluorescence emission band. As it was discussed above, the decay of the $S_{1}$ state is controlled dominantly either by the dipole–dipole Förster energy transfer mechanism (proportional to the overlap of both spectra) or by the exciton hopping transport followed by the exciton recombination when two excitons reach adjacent molecules. This recombination might take place by Dexter mechanism, which also requires overlapping absorption (S_1_–S_n_) and emission (S_1_–S_n_) spectra. Due to the significant overlap between the ESA spectrum of the state $S_{1}$ , and its luminescence spectrum found by Rais et al. (2017b), we can conclude that upon the $S_{1}-S_{1}$ “collision”, the reaction pathway $|S_{1},S_{1}\rangle \to |S_{n},S_{0}\rangle$ will be efficient (see Figure 6A). The obtained energy 3.79 eV of the higher excited state $S_{n}$ at the relaxed $S_{1}$ geometry will be only slightly higher than the maximum energy 3.65 eV of the direct vertical absorption $|S_{0}\rangle \to |S_{n}\rangle$ to the higher excited state (see Figure 6B). The relaxation kinetics of this manifold was also directly mapped in the study by Rais et al. (2017b) by the excitation at 3.73 eV. It was shown that during ca 100 fs after such photoexcitation, the ESA spectrum becomes almost identical to the ESA spectrum of the state $S_{1}$ obtained directly by the excitation at 2.82 eV. It shows that the internal conversion by the Kasha process $|S_{n}\rangle \to |S_{1}\rangle$ is achieved during ca 100 fs. We note that the green and violet arrows in Figure 6A only indicate the overlap of ESA and steady-state luminescence spectra. However, as the Förster energy transfer is the resonance process with only “virtual” absorption and luminescence on respective unimers, no luminescence during the singlet–singlet annihilation is present. In the study by Rais et al. (2017b), also a singlet fission (SF) was observed that occurred within ca 160 fs after photoexcitation (see Figure 6B). It shows that the *S*_2_ singlet relaxes by the Kasha process until it reaches energy resonance with triplet pairs $T_{1}T_{1}$ . It means that upon the collision reaction $|S_{1},S_{1}\rangle \to |S_{n},S_{0}\rangle$ , both the Kasha and SF processes can take place. Since the hot ballistic energy is released and redistributed to the vibrational manifold during the Kasha process, it seems reasonable to assume that the energy of the hot triplet pair state exceeds its binding energy, and the triplet pair can dissociate, that is, the reaction $T_{1}T_{1}\to T_{1}+T_{1}$ takes place. The rate of the triplet pair dissociation was shown to be strongly dependent on the temperature (Stern et al., 2017) so that very hot local temperature during the Kasha relaxation followed by the subsequent SF process can finally produce two separated triplet states. However, at earlier stages, before the correlated triplet pairs fully dissociate, the corresponding ESA spectrum may slightly differ from that of uncorrelated triplet pairs. This can also potentially explain why the triplet kinetics obtained by the two methods are different at early times, too.

Assuming singlet–singlet annihilation, we can derive the rate G(t) of triplet generation

$$G\left(t\right)=\beta \gamma \left(t\right)S{\left(t\right)}^{2}=-\beta \frac{\partial }{\partial t}S\left(t\right)$$ (16)

and

$$\overset{t}{\underset{0}{\int }}d\tau G\left(\tau \right)=\beta \left(S\left(0\right)-S\left(t\right)\right).$$ (17)

Thus, the decrease in the generation rate of triplets by the singlet–singlet annihilation can be obtained by analyzing the experimental data of the time evolution of (S(0)−S(t)) .

As observed from the previous two paragraphs, by comparing the rates of saturation of $\overset{t}{\underset{0}{\int }}S\left(\tau \right)d\tau$ and (S(0)−S(t)) , that are related to the triplet formation either by the inter-system crossing or singlet–singlet annihilation, with the integral $\overset{t}{\underset{0}{\int }}G\left(\tau \right)d\tau$ obtained from experimental data, we can distinguish between the inter-system crossing and singlet–singlet mechanisms.

**FIGURE 7:**
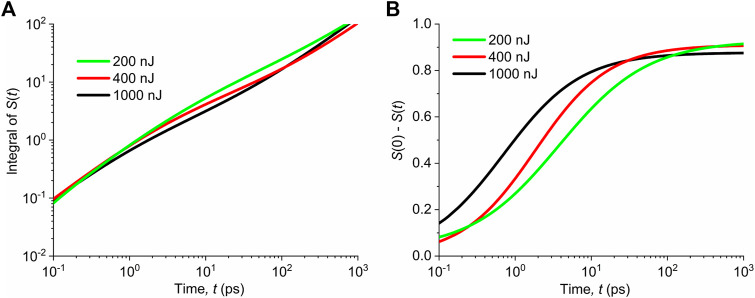
**(A)** Calculated dependences of $\overset{t}{\underset{0}{\int }}d\tau S\left(\tau \right)$ (triplet population generated by the inter-system crossing). **(B)** Calculated dependences of S(0)−S(t) (triplet population generated by the singlet bi-molecular annihilation) from the fitted singlet population S(t) . Different intensities of pump fluences are shown in the legend. Initial singlet population is normalized to unity.

The dependences of $\overset{t}{\underset{0}{\int }}S\left(\tau \right)d\tau$ obtained from the fitted experimental data of the singlet exciton population for different pump fluences (see Figure 2) are shown in Figure 7A, normalized to the initial singlet exciton populations. We can see that the time profiles of the function $\overset{t}{\underset{0}{\int }}S\left(\tau \right)d\tau$ are very similar, practically independent on the pump fluence, and that they obey the power-law dependence $∼t^{-0.75}$ , with no observable saturation for all pump fluences.

It indicates that at the initial rise times (rate of formation), the triplet states are not governed by the singlet-to-triplet inter-system crossing.

The time evolutions of (S(0)−S(t)) are plotted in Figure 7B for different pump fluences, again normalized to unity, that is, S(0)=1 . We see that these dependences saturate in time, depending on the pump fluence. With increasing pump fluence, the initial rise time is shorter. It suggests that the triplets are formed by singlet–singlet annihilation mechanism.

It should be noted that the dependences in Figure 7 are obtained by numerical fitting of the experimental data. The saturation of (S(0)−S(t)) at the values near 0.9, not reaching 1 as it would be expected, is caused by the limited experimental time window of 6 ns, which did not allow the singlets to be fully de-excited., for example, by luminescence. It also indicates that the linear decay terms in Eq. 3 could be neglected on this time window and, thus, the singlet population decay is controlled mainly by the bimolecular annihilation.

**FIGURE 8:**
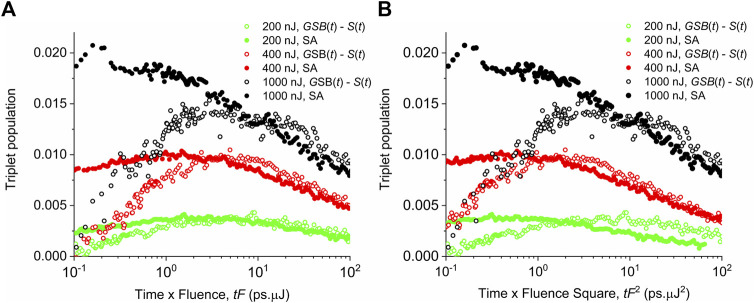
Experimental data of the triplet exciton population evolution for different pump fluences F shown in the legend. Empty symbols—data obtained by subtracting the ground-state bleach and the singlet populations. Full symbols—data from the spectral analysis (SA) of transient absorption reported by Rais et al., 2017b. In *x*-axis, time is rescaled as tF **(A)**, and $\;tF^{2}$ **(B)**.

We can also compare the different mechanisms of the triplet formation looking at the explicit dependence of the rise time (or maximum position) of the triplet population on the pump fluence *F*, that is, on the initial singlet population S(0) . The experimental data of the triplet population are plotted in Figure 8 against tF (A) and $tF^{2}$ (B). Although the dependences plotted in the log–log scale are less sensitive to the linearly or quadratically increasing value of F , we can still see general trends. For the former case (Figure 8A), we see that the maximum of the peaks shifts systematically to the left with increasing pump fluence F if the triplet populations are obtained either from the spectral analysis ESA or by the subtraction of the ground-state bleach and singlet populations. On the other hand, for the latter case (Figure 8A), we cannot see any systematic shift of the peak position with increasing pump fluence for the data obtained by both methods. In this case, we rather see some uncertainty in the peak position. Findings in Figure 8 promote the scaling of the triplet population rise time rather with $tF^{2}$ $\left(tS{\left(0\right)}^{2}\right)$ than with tF (tS(0)) . This is another supporting argument that the triplets were formed by the singlet–singlet annihilation process.

**FIGURE 9:**
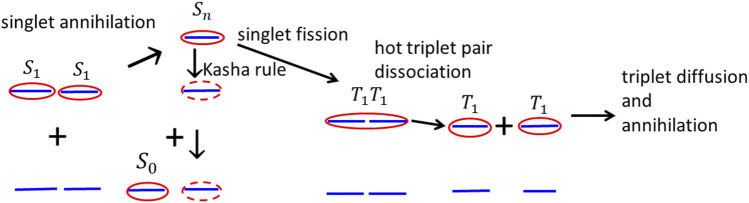
Schematic figure of subsequent processes of mutual singlet exciton annihilation, formation of intermediate hot high excited singlet exciton, formation of the hot intermediate triplet exciton pair, its dissociation to two triplet states, triplet state diffusion, and triplet exciton–exciton annihilation.

Based on the analysis of Figures 7, 8, we conjecture that triplets are created by the singlet–singlet annihilation rather than by inter-system crossing. Hot individual triplet excitons migrate by diffusion and gradually thermalize until they finally collide and annihilate. The whole process of singlet–singlet annihilation, triplet pair formation and dissociation, and triplet diffusion and annihilation is schematically drawn in Figure 9. The triplet–triplet annihilation in long times can also potentially contribute to the formation of singlet states $S_{1}$ as these states have lower energy than the bound triplet pair states. It can possibly explain the residual singlet population at longer times seen in Figure 2, expressed also as the additive term in Eq. 2.

### Triplet Diffusion and Annihilation

We can, thus, assume that the evolution of the triplet population is controlled simultaneously by their generation from singlet states and by the triplet–triplet annihilation. The first process dominates at early stages, as it was discussed above, while the triplet–triplet annihilation becomes dominant at longer times. As a supporting argument, we can point to the sensitivity of the triplet population decay rate to the pump fluence at longer times, seen in Figure 5, which is in accordance with the mutual collisions of triplet excitons. These long-time tails of the triplet population decay show the power-law dependence, which is typical for the bi-molecular collisions, too. The kinetics of the triplet population can, thus, be described by the equation

$$\frac{\partial }{\partial t}T\left(t\right)=-\beta \frac{\partial }{\partial t}S\left(t\right)-\gamma_{1d}^{T}\left(t\right)T{\left(t\right)}^{2}.$$ (18)

The triplet–triplet annihilation rate $\gamma_{1d}^{T}\left(t\right)$ is controlled by 1d diffusion of triplet excitons *via* unimer units. We fitted the experimental data of the triplet exciton population decay according to Eq. 18, taking $\gamma_{1d}^{T}\left(t\right)$ as an “ansatz.” The results given in Figure 5 show that the decay takes a power-law dependence. As the formation of “free” triplets proceeds through the formation of intermediate higher singlet excitons and triplet pair states, the power-law ansatz for $\gamma_{1d}^{T}\left(t\right)$ at early times may not be adequate. However, at times longer than 10 ps, free triplets are thermalized and the triplet exciton population evolution will be controlled by the diffusion of mutually independent species. Here, we fitted the experimental data obtained by the spectral analysis method and did not use data from the difference between the ground-state bleaching and singlet populations because at various stages of intermediate transitions within the excited states manifold, the population might not be conserved (see Figure 9). After the rate $\gamma_{1d}^{T}\left(t\right)$ has been obtained, we can also determine the 1d diffusion coefficient $D_{1d}^{T}\left(t\right)$ of triplets similarly as in the case of singlets (cf. Eq. 8).

$$D_{1\text{d}}^{\text{T}}\left(t\right)=\frac{\pi }{16}a^{2}\gamma_{1d}^{T}\left(t\right)\overset{t}{\underset{0}{\int }}\gamma_{1d}^{T}\left(\tau \right)d\tau .$$ (19)

**FIGURE 10:**
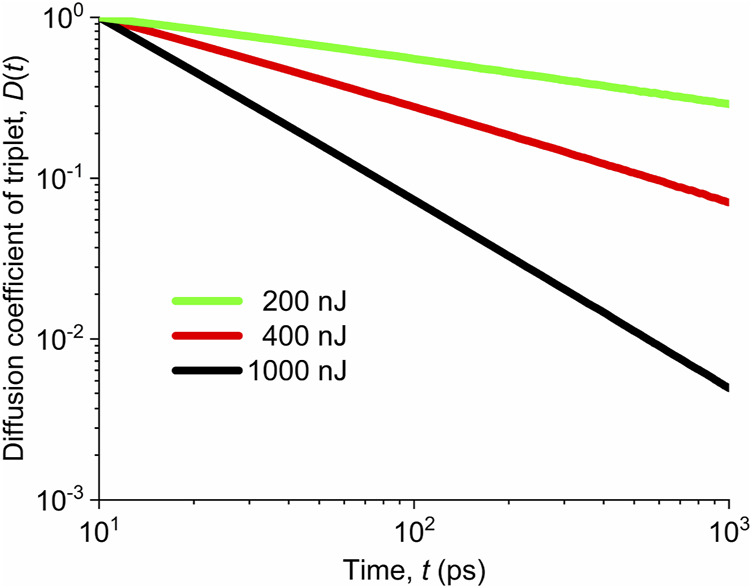
Normalized values of the diffusion coefficient of triplets for different pump fluences marked in the legend.

The time dependences of the normalized 1d diffusion coefficient $D_{1d}^{T}\left(t\right)$ of triplets are plotted in Figure 10 for various pump fluences.

We did not show the diffusion coefficient $D_{1d}^{T}\left(t\right)$ in absolute values for several reasons: First, at early times, the evolution of triplets is strongly proportional to the decay rate of singlets. This rate was obtained by fitting the experimental data, which brings some numerical uncertainty. Second, at short times, the triplet population consists dominantly of hot intermediate triplet pairs and not of individual triplets. Using the power-law ansatz for $\gamma_{1d}^{T}\left(t\right)$ can then bring some systematic deviation at early times. Third, the absolute values of triplet exciton populations in Eq. 18 are obtained *via* spectral decomposition of transient differential absorbance data projected to the basis spectral functions of singlets and triplets. As the population of singlets is significantly higher than that of triplets, a potential error of such decomposition has impact on the accuracy of the triplet exciton population. If such triplet populations T(t) are exact up to a factor f , the kinetic rates $\gamma_{1d}^{T}\left(t\right)$ are exact up to the factor 1/f due to the bilinear nature of the triplet exciton decay in Eq. 18. The diffusion coefficient $D_{1d}^{T}\left(t\right)$ obtained *via* Eq. 19 is then exact up to the factor $1/f^{2}$ . If we compare the evolutions of the normalized diffusion coefficient of triplet states for different pump fluences, we clearly see the power-law decrease of the diffusion coefficient with time, that is, $D_{1d}^{T}\left(t\right)∼t^{-p}$ .

For the pump fluences around 200 nJ, the diffusion coefficient $D_{1d}^{T}\left(t\right)$ only slowly changes with time, but it decreases faster under increased pump fluence. Estimated values of the exponent p for different pump fluences are summarized in Table 2. We see that the exponent p scales almost linearly with the pump fluence.

**TABLE 2:** Dependence of the exponent p in the expression $D_{1d}^{T}\left(t\right)∼t^{-p}$ for the diffusion coefficient of triplets on the pump fluence F .

Pump fluence F[nJ]	Exponent *p*
200	0.26
400	0.58
1,000	1.15

The correlation between the power-law time dependence of the exciton population and the time-dependent diffusion coefficient was proved theoretically and experimentally documented on thin films of α,ω-bis(tpy)terthiophene (T) unimers without metal couplers under strong excitation conditions (Menšík et al., 2019). The power-law diffusion kinetics can generally occur in systems with an increased structural and dynamic disorder (see Devižis et al., 2009; Král and Menšík, 2016; Toman et al., 2017; Menšík et al., 2018b and references therein). In the system discussed in this article, the structure can be disturbed by both the electronic excitations and heat dissipation at stronger pump fluence which, subsequently, can affect triplet diffusivity.

## Conclusion

The detailed analysis of the kinetics of the photoexcited states in thin films of metallo-supramolecular polymers with ditopic thiophene-bridged terpyridine ligands showed that singlet excitons $S_{1}$ are formed upon photoexcitation at the wavelength 440 nm. The pump fluence dependent rate of the $S_{1}$ singlet exciton population decay proved the exciton–exciton annihilation nature of the decay mechanism. Our theoretical modeling showed that the exciton–exciton annihilation process is controlled more by the Förster energy transfer of singlets than by their diffusion. We have also shown that mutual collisions of singlet excitons can partially yield triplet excitons *via* singlet fission of higher excited singlet states formed within these collisions. It explains the dependence of the rate of triplet state formation on the pump fluence. We have shown that the triplet state population decays *via* mutual annihilation and found that the diffusion coefficient follows the power-law time dependence $t^{-p}$ with the exponent p increasing almost linearly with the pump fluence. The faster decrease of the diffusion coefficient with time for higher pump fluence was assigned to the increased disorder due to the dissipated excitation energy.

The reported model is generally applicable to systems in which the energy of triplet pairs falls within the energy levels of S_1_ and S_2_ states. In systems where the triplet diffusion is slower, the excitation energy can be temporarily stored in these triplets and can be restored as S_1_ energy later, which is manifested by prolonged lifetime of S_1_ excitons, observable, for example, as delayed fluorescence.

## Data Availability

The raw data supporting the conclusion of this article will be made available by the authors, without undue reservation.
